# Some General Considerations as to the Various Methods of Treatment to Be Adopted in Skin Affections

**Published:** 1894-03-03

**Authors:** H. W. Marett Tims

**Affiliations:** Lecturer on Biology and Comparative Anatomy at the Westminster Hospital Medical School, and Clinical Assistant at the Hospital for Diseases of the Skin, Blackfriars


### Medical Progress and Hospital Clinics.

[*The Editor will be glad to receive offers of co-operation and contributions from members of the profession. All letters should be addressed to* The Editor, The Lodge, Porchester Square, London, W.]

### SOME GENERAL CONSIDERATIONS AS TO THE VARIOUS METHODS OF TREATMENT TO BE ADOPTED IN SKIN AFFECTIONS.

By H. W. Marett Tims, M.D.Edin., Lecturer on Biology and Comparative Anatomy at the Westminster Hospital Medical School, and Clinical Assistant at the Hospital for Diseases of the Skin, Blackfriars.

Before entering into a detailed consideration of each individual affection of the skin, it is well to have clearly in mind some of the general principles upon which the treatment may rationally be based, and it is to these general principles that I wish in the first instance to draw attention.

They may be broadly divided into three groups—General, Local, and Operative.

(1) *General.*—Before attempting to treat a skin affection, it is essential that the general health of the patient should be inquired into, as very many are distinctly traceable to derangements of the various functions of the internal organs. To give a few of the more prominent examples. One of the commonest skin diseases with which we have to deal is eczema, and how often do we find it associated with gout or rheumatism, or even attacks of eczema alternating with spasmodic asthma? Or, again, the patient with eczema is often delicate and weakly, and in evident need of tonic treatment.

Acne, in all its forms, is most frequently associated with flatulent dyspepsia and constipation. To treat the acne successfully the state of the stomach and bowels must be attended to.

An eminent dermatologist has said, in answer to the question, What diseases he was most frequently called upon to treat? "Diseases of the alimentary canal."

And here I may say, with regard to dieting, that, beyond the general principles of dieting in dyspepsia, all highly-spiced foods and condiments, all salt meats and fish, and all stimulants are, as a general rule, contra-indicated.

Syphilitic affections of the skin, too, are unfortunately very common, and no amount of external application will be of any avail without the aid of internal treatment.

It will hardly be necessary to point to such hygienic conditions as cleanliness and fresh air; these are essential in every case, but must not be pushed to extremes. To explain my meaning: We often see cases of chronic eczema of the palms in which the skin is hard, dry, and cracked, or affections in which there is active hyperæmia. In both these conditions the frequent use of water is not only painful but distinctly injurious, so that the process of washing should be limited to once a day, and then done as rapidly as possible.

In a large number of cases, for the removal of scales and of offensive secretions, and for the relief of irritation, baths are distinctly useful, and their value may often be enhanced by the addition of substances so that they become both palliative and curative as well as cleansing.

Before leaving the general treatment, I must not omit the importance of rest, even in bed, in many affections, more especially in those whose onset is accompanied by fever. No sketch. however brief, would be complete without mentioning the use of thyroid extract. The improvement in the skin which took place during the treatment of cases of myxœdema with thyroid gland led Dr. Byrom Bramwell to adopt it in cases of psoriasis with most markedly beneficial results. I must confess my experience in about thirty cases has been disappointing; nevertheless, in some few instances it has proved of some use. The time is too short since this remedy was first tried to pronounce any judgment, but the results so far seem to be either very good or nil, so that one is led to ask, Are there two clinical varieties of psoriasis, distinct also as to their origin?

(2) *Local.*—External applications may be in the form of ointment, powders, poultices, pigments, and lotions or liniments, and of these poultices are of but little use. They are certainly soothing in inflamed conditions, but I think the value of them is not nearly so great as many other applications. In my own practice I limit their use to the removal of crusts in impetiginous conditions.

Where there is active hyperæmia I prefer lotions, and if this condition goes on to "weeping," as in acute eczema, dusting with powder, for example kaolin, oxide or oleate of zinc, and many others, is useful. In the very acute stages I have seen very excellent results from painting the part over with some of the so-called jellies, which are of various kinds, as of zinc and icthyol, or zinc and glycerine. Of course the most common of all outward applications are ointments, and they vary in their action, astringent, stimulating, caustic, &c., according to their composition; but here again I may add that I do not think they are so good, even the mildest of them, in acute hyperæmic states as lotions. Bandaging in all conditions is valuable in giving support to the weakened tissues.

(3) *Operative.*—Skin affections requiring anything of the nature of an operation are few. Dermatology is, so far, the one special branch not entirely monopolised by the surgeon, though they have lately shown signs of further encroachment on this medical preserve.

Lupus is the affection which most commonly calls for treatment of this nature, and it may be in the form of linear scarification, scraping, or the more recently-adopted plan of excision. Of the first two, I consider scraping to be preferable as more likely to get rid of all the diseased tissue. The results obtained by Mr. Watson Cheyne and by Mr. Bidwell by excision have so far been most excellent. Some of these cases I have had an opportunity of seeing, and I think it is a method of treatment of great value. It remains to be seen whether the ultimate result is as good or better than scraping, but *prima facie* one may expect it to be better.

The next most common disease calling for this kind of treatment is rodent ulcer, and in these cases early and complete removal is the only treatment justifiable.

Electrolysis may also be mentioned here. It is useful in some cases of nævus, and has been used in lupus, but is not, I believe, much used now.

The object in reviewing briefly the general considerations as to treatment is to draw attention to the importance of the general treatment in conjunction with the local. In former times the internal treatment —"the cooling the blood"—was almost exclusively adopted, and now there seems to be a tendency to go to the other extreme and use local treatment only, while the general treatment is omitted.

Are not the number of "specifics" which are constantly brought out, and have their little day and then pass into the realms of oblivion, signs of the times?

To be successful in the treatment of skin affections the two must be considered. In certain cases one is of more importance than the other, but in few, if any, can either be ignored.

